# An integrative histopathologic clustering model based on immuno‐matrix elements to predict the risk of death in malignant mesothelioma

**DOI:** 10.1002/cam4.3111

**Published:** 2020-05-11

**Authors:** Marcelo Luiz Balancin, Walcy Rosolia Teodoro, Cecilia Farhat, Tomas Jurandir de Miranda, Aline Kawassaki Assato, Neila Aparecida de Souza Silva, Ana Paula Velosa, Roberto Falzoni, Alexandre Muxfeldt Ab'Saber, Anja C. Roden, Vera Luiza Capelozzi

**Affiliations:** ^1^ Department of Pathology Faculdade de Medicina da Universidade de São Paulo São Paulo Brazil; ^2^ Rheumatology Division Hospital das Clinicas da Faculdade de Medicina da Universidade de São Paulo São Paulo Brazil; ^3^ Department of Laboratory Medicine and Pathology Mayo Clinic Rochester MN USA

**Keywords:** biomarkers, cluster analysis, collagen type V, computational pathology, extracellular matrix, immunomodulation, mesothelioma

## Abstract

**Objective:**

Previous studies have reported a close relationship between malignant mesothelioma (MM) and the immune matricial microenvironment (IMM). One of the major problems in these studies is the lack of adequate adjustment for potential confounders. Therefore, the aim of this study was to identify and quantify risk factors such as IMM and various tumor characteristics and their association with the subtype of MM and survival.

**Methods:**

We examined IMM and other tumor markers in tumor tissues from 82 patients with MM. These markers were evaluated by histochemistry, immunohistochemistry, immunofluorescence, and morphometry. Logistic regression analysis, cluster analysis, and Cox regression analysis were performed.

**Results:**

Hierarchical cluster analysis revealed two clusters of MM that were independent of clinicopathologic features. The high‐risk cluster included MM with high tumor cellularity, high type V collagen (Col V) fiber density, and low CD8^+^ T lymphocyte density in the IMM. Our results showed that the risk of death was increased for patients with MM with high tumor cellularity (OR = 1.63, 95% CI = 1.29‐2.89, *P* = .02), overexpression of Col V (OR = 2.60, 95% CI = 0.98‐6.84, *P* = .04), and decreased CD8 T lymphocytes (OR = 1.001, 95% CI = 0.995‐1.007, *P* = .008). The hazard ratio for the high‐risk cluster was 2.19 (95% CI = 0.54‐3.03, *P* < .01) for mortality from MM at 40 months.

**Conclusion:**

Morphometric analysis of Col V, CD8^+^ T lymphocytes, and tumor cellularity can be used to identify patients with high risk of death from MM.

## Graphical abstract

Malignant mesothelioma (MM) and the immune matricial microenvironment (IMM). Hierarchical cluster analysis revealed two distinct clusters of patients with either low or high risk for death of MM. Collagen V, CD8^+^ T lymphocytes and tumour cellularity densities characterized high and low risk subgroups with impact on outcome.

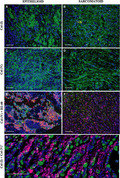

## 1 INTRODUCTION

Malignant mesothelioma (MM) is a highly lethal disease arising from mesothelial cells of the pleural, peritoneal, and pericardial cavities. The very low 5‐year survival rate of 16.6%^1^ has not been improved over decades despite extensive research, indicating that a better and more detailed understanding of this type of cancer has to be achieved in order to design effective treatment strategies. The diagnosis of MM is complex because of various epithelial and/or mesenchymal patterns, its phenotypic variability from patient to patient, and its property of mimicking other cancers, particularly adenocarcinoma or benign processes, which may lead to a delay of the diagnosis or inappropriate treatment.^2^ The histologic classification of MM includes three major subtypes: epithelioid (~60% of cases), sarcomatous (~20% of cases), and biphasic (~20% of cases).^3^ At present, histological subtype, lymph node status, and pathological TNM stage are prognostic factors for overall survival (OS).^4^, ^5^, ^6^ Patients diagnosed with epithelioid mesothelioma have the longest survival (12‐27 months). The appropriate course of treatment of MM can be difficult to determine^7^, ^8^ and currently is largely guided by histological subtype, clinical stage, and patient characteristics rather than objective biomarker signatures. Biomarkers that could assist in more individualized treatment are much needed. Thus, there is great interest in the development of biomarkers that might help to identify which MMs are likely to progress and lead to shorter survival of the patient.^9^

Autoimmune responses have been associated with asbestos exposure to induce antitumor immunity by CD8^+^ cytotoxic T‐lymphocyte cells and extracellular matrix (ECM) synthesis.^10^, ^11^ The interaction between CD8^+^ cytotoxic T‐lymphocyte cells and the ECM to identify mechanisms that might relate to tumor progression and shortened survival has been studied mainly in small and heterogeneous series and the data are inconclusive.^12^, ^13^, ^14^, ^15^ Because the ECM plays a key regulatory role through a network of signaling molecules that alter the malignant potential of cells and consequently their invasive growth, quantitative and qualitative modifications of the collagen content of the ECM have been targeted as potentially useful tumor markers.^16^, ^17^, ^18^, ^19^, ^20^, ^21^, ^22^ In MM, a pro‐tumorigenic role of collagen type I (Col I) has been described,^23^ whereas the function of type V collagen (Col V) has not yet been thoroughly studied. Col V is a regulatory fibril‐forming collagen which comprises only 2‐5% of the total collagens in normal tissues. It has been shown that Col V‐mediated activation of the β1‐integrin signaling pathway promotes cell migration and motility.^24^ It has also been reported that Col V overexpression may lead to the development of anti‐Col V immunity facilitating tumor progression.^25^

In this study, we explored the quantitative relationship between Col V and CD8^+^ T lymphocytes and outcome of 82 patients with MM as well as their relationship with the tumor and tumor microenvironment factors. We first assessed the expression of several tumor and microenvironmental markers in epithelioid MM and sarcomatoid MM. Second, we explored the quantitative similarities between these factors by clustering to characterize a low‐ and high‐risk subgroup of MM. Finally, we tested the impact of the two subgroups on patient outcome.

## 2 MATERIALS AND METHODS

### 2.1 Case selection and clinicopathological review

In this retrospective study, patients with MM were retrieved from hospital records (2008‐2019). The study was approved by the Ethics Committee of our Institution. Demographic and clinical data including follow‐up data were obtained from medical records. All original slides were reviewed by two expert pathologists and classified according to the 2015 World Health Organization Classification of Pleural Tumors.^3^ Tumor‐infiltrating lymphocytes (TILs) were scored according to a three‐tier scoring system for solid tumors, as previously proposed^26^: score 1 (TILs occupy less than 10% of tumor area), score 2 (10%‐50%), and score 3 (more than 50%) exclusively in the intratumoral component. Nuclear grade was classified according to the guidelines proposed by Kadota and colleagues^27^ (nuclear atypia, nuclear/cytoplasmic ratio, chromatin pattern, intranuclear inclusions, prominence of nucleoli, mitotic count, and atypical mitoses) and classified in a three‐tier system.

### 2.2 Tissue microarray, histochemistry, immunohistochemistry, and in situ hybridization

Tumor tissues were fixed in 10% buffered formalin and embedded in paraffin. Representative tumor blocks were selected after a review of all the original glass slides. Three 1.0‐mm cores per case were annotated and extracted from the center of tumor areas, providing a triplicate sampling of all cases, totaling 229 valid cores for scoring (70% of cores) in a total of three recipient tissue microarray (TMA) blocks.

The slides were stained with a standard hematoxylin and eosin (H&E) staining protocol and with the modified Russell‐Movat protocol,^28^ mixing five stains (alcian blue, Verhoeff's hematoxylin, and Crocein Scarlet combined with acidic fuchsine and saffron at pH 2.5).

Table S1 shows the primary antibodies, clones, dilution, and source used for immunohistochemistry. Immunohistochemical staining was performed to evaluate immune cells (CD4, CD8, CD20, and CD68), mismatch repair proteins (MLH1, PMS2, MSH2, and MSH6), mesothelial cells (WT1, D2‐40, MOC31, and BerEP4), cellular characteristics (Ki‐67 and p53), and potential therapeutic targets (PD‐1, PD‐L1, and CD30). The 4‐µm thick TMA sections were subjected to immunohistochemistry staining with manual and automated techniques. For the manual technique, the Novolink Max Polymer (Novocastra, Newcastle upon Tyne, UK), pressure cooking antigen retrieval, biotinylated rabbit anti‐mouse immunoglobulin G (Dako, Carpinteria; dilution 1:400), streptavidin combined in vitro with biotinylated horseradish peroxidase (Dako; dilution 1:1000), diaminobenzidine tetrahydrochloride, and counterstaining with hematoxylin were used. The automated technique was performed on a Ventana Benchmark Ultra Platform (Roche, Ventana Medical Systems Inc, Tucson, USA) with the OptiView DAB IHD Detection Kit and OptiView Amplification Kit with proprietary protocols. Immunohistochemistry (IHC) staining proteins were evaluated using a membrane algorithm to analyze immune checkpoint inhibitors, and a cytoplasmic algorithm to analyze immune cells, MOC31, BerEP4 and D2‐40. P53, WT1 and Ki‐67 proteins were assessed using nuclear algorithm, which counted cells positive for expression. PD‐L1 was scored as tumor proportion score (number of PD‐L1‐positive tumor cells divided by total number of tumor cells multiplied by 100).

In situ hybridization was employed to identify apoptosis via the nuclear expression of the tumor and surrounding environment by the terminal deoxynucleotidyl transferase (TdT) method of nick‐end labeling (TUNEL) (Boehringer Mannheim, Mannheim, Germany). This method involves the addition of deoxyuridine triphosphate labeled with fluorescein to the ends of the DNA fragments by the catalytic action of TdT. Paraffin‐embedded sections (3‐4 µm) were layered onto glass slides. The tissue sections were dewaxed with xylene and rehydrated with graded dilutions of ethanol in water. The sections were then washed four times with double distilled water for 2 minutes and immersed in TdT buffer (Boehringer Mannheim). Next, the sections were covered with TdT (0.3 U/µL) and fluorescein‐labeled deoxyuridine triphosphate in TdT buffer and then incubated in a humidified atmosphere at 37°C for 60 minutes. For negative controls, TdT was eliminated from the reaction mixture. The sections were then incubated with an antibody specific for fluorescein conjugated to peroxidase. The staining was observed with a substrate system, in which nuclei with DNA fragmentation were stained brown. The reaction was terminated by washing the sections twice in phosphate‐buffered saline (PBS). The nuclei without DNA fragmentation were stained blue as a result of counterstaining with hematoxylin.

### 2.3 Immunofluorescence

To perform immunostaining for Col I and Col V, 3‐µm paraffin‐embedded sections were mounted on 3‐aminopropyltriethoxysilane (Sigma Chemical Co.), dewaxed in xylene, and hydrated in graded ethanol. Antigen retrieval was determined by the enzymatic treatment of tissue with bovine pepsin (10 000 dry units/ml; Sigma Chemical Co.) in acetic acid buffer at 0.5 N (pH 2.2) (4 ng/mL) for 30 minutes at 37°C. Nonspecific sites were blocked with 5% bovine serum albumin (BSA) in PBS for 30 minutes at room temperature. The slides were then incubated overnight with rabbit polyclonal anti‐human type I (1:200) and V (1:300) collagen (Rockland). Additionally, double‐type polyclonal anti‐human type V collagen (1:300) and D2‐40 (1:1000) (Dako) staining were performed. For negative controls, the sections were incubated with fetal bovine serum instead of the primary antibody. The same treatment was carried out for immunofluorescence detection. The sections were incubated with the same primary antibody diluted with PBS plus BSA 1% overnight at 4°C in a humidified atmosphere. The sections were then washed in PBS with Tween_20_ 0.05% and incubated for 60 minutes at room temperature with Alexa 488‐conjugated goat anti‐mouse IgG (1:200, Invitrogen) and Alexa 546‐conjugated goat anti‐rabbit IgG (1:200, Invitrogen). The nuclei were counterstained with 0.4 mmol/L/mL 4′,6‐diamidino‐2‐phenylindole, dihydrochloride (DAPI; Molecular Probes^TM^, Invitrogen) for 15 minutes at room temperature. For negative and autofluorescence controls, the sections were incubated with PBS and normal rabbit or mouse serum instead of the specific antibody. Specimens were mounted with an aqueous mounting medium and observed using immunofluorescence microscopy (Olympus BX51, Olympus). Image analysis was used to quantitate different types of collagen fibers (I and V) in the tumor microenvironment.

### 2.4 Image analysis and morphometry

All brightfield‐observed slides were scanned using the Panoramic 250 whole slide scanner (3DHistech, Budapest, Hungary) at ×40 magnification. Stained TMA sections were disarrayed within QuPath version 0.2.0‐m4^29^, ^30^ (Centre for Cancer Research & Cell Biology, University of Edinburgh), an open‐source image analysis platform. All cores were evaluated during the scoring process to manually exclude invalid cores (less than 10% of tumor per core or artifacts).

For TMA quantification on QuPath, a simple, automated semi‐assisted method was employed. A desired threshold for positive cells was chosen for each marker after a few steps and posterior validation. Staining vectors were automatically analyzed for each scanned TMA slide, followed by total tissue area detection, separation of tumor from non‐tumor areas in each core, and finally, automatic cellular detection. Positive cells were assigned through the optic density threshold from selected cells, tested on each core and after validation by an expert pathologist, applied to the whole array. For each marker, a threshold was employed after a manual trial‐and‐error fashion. For these data, the results were established as the number of positive cells per mm^2^ of tissue and exported from the software. A corresponding script was then generated and executed on all imported TMA slides from each marker, automating the detection and exportation steps across all slides.

For Movat pentachrome staining quantification, the machine learning Trainable Weka (Waikato Environment for Knowledge Analysis Segmentation) plugin^31^ was used in ImageJ (National Institute of Health). A training set was created by a pathologist from 20 10 × 10 titles compiled from the ground truth areas of all slides. Reticulin, elastin, collagen, cells, hyaluronic acid, and background were assigned to separate monochrome colors (red, black, yellow, purple, blue, and pink, respectively) for an 8‐bit segmented image. Color‐specific thresholds were applied and generated 8‐bit monochrome images per component for quantification. The data were extracted as a percentage of expression per mm^2^.

For fluorescent images, a low‐power image of the TMA core was acquired followed by four 40× images of all filters of interest through an Olympus camera (Olympus Co., St. Laurent) coupled to an Olympus microscope (Olympus BX51) using Image‐Pro Plus 6.0 software (Media Cybernetics). All high‐power fluorescence images were segmented through selective optical threshold densities on Image‐Pro Plus, and the data were extracted as collagen constitution per mm^2^.

All computational pathology was performed on an off‐the‐shelf Intel Core i7 6700K with 32 GB RAM and 512 GB SSD running the mentioned software on Windows 10 (Microsoft Corporation).

### 2.5 Statistical analysis

Statistical analysis was performed using SPSS v18 (Chicago, IL, USA) for Windows. The relationships between quantitative variables were evaluated by Student's *t* test, and analysis of variance was used to correlate staining patterns. Multivariate analysis was carried out using the Cox proportional hazards ratio. In addition, paired‐samples *t* test and general linear model were used to test the relationship between one continuous variable and several others. A two‐tier cluster model was elaborated in a semi‐assisted fashion using the hierarchical clustering feature, and significant factors were selected based on the multivariate regression model and biological significance, integrating cellular, immunologic, and matrix elements. Multiple models were generated, and the most significant model that integrated the desired elements and correlated with the survival endpoint was chosen. The cumulative OS time was calculated by the Kaplan‐Meier method and analyzed by the log‐rank test. For all tests, a two‐tailed p‐value of less than 0.05 was considered statistically significant.

## 3 RESULTS

The clinical and histologic characteristics of the patients are summarized in Table 1. All patients were of stage III or IV. The median follow‐up time of the patients was 22 and 18 months for epithelioid and sarcomatoid histotypes, respectively. Forty‐nine (56%) patients died of recurrence or metastases.

**Table 1:** Clinical characteristics of patients with malignant mesothelioma

Characteristic	Epithelioid (N = 73)	Sarcomatoid (N = 9)	*P* value
Age, median	60 y	56 y	.51
Sex, n (%)			
Male	52 (63%)	6 (7.3%)	.52
Female	21 (25.6%)	3 (3.7%)	
Asbestos exposure^a^, n (%)			
No	35 (42.7%)	3 (3.7%)	.32
Yes	38 (46.3%)	6 (7.3%)	
Site of malignant mesothelioma, n (%)			
Pleura	54 (65.9%)	8 (9.8%)	.57
Extrapleural	19 (23.2%)	1 (1.2%)	
Stage^b^ III/IV	73 (100%)	9 (100%)	
Specimen type			
Biopsy	11 (13.4%)	1 (1.2%)	.60
Surgical resection	62 (75.6%)	8 (9.8%)	
Treatment, n (%)			
Surgery	62 (75.6%)	8 (9.8%)	.54
Chemotherapy	13 (15%)	2 (2.3%)	.55
Follow‐up, median	22 mo	11 mo	.56

^a^ Asbestos unknown: nine epithelioid MM.

^b^ Per International Association for the Study of Lung Cancer (IASLC) criteria.^31^

**Figure 1:**
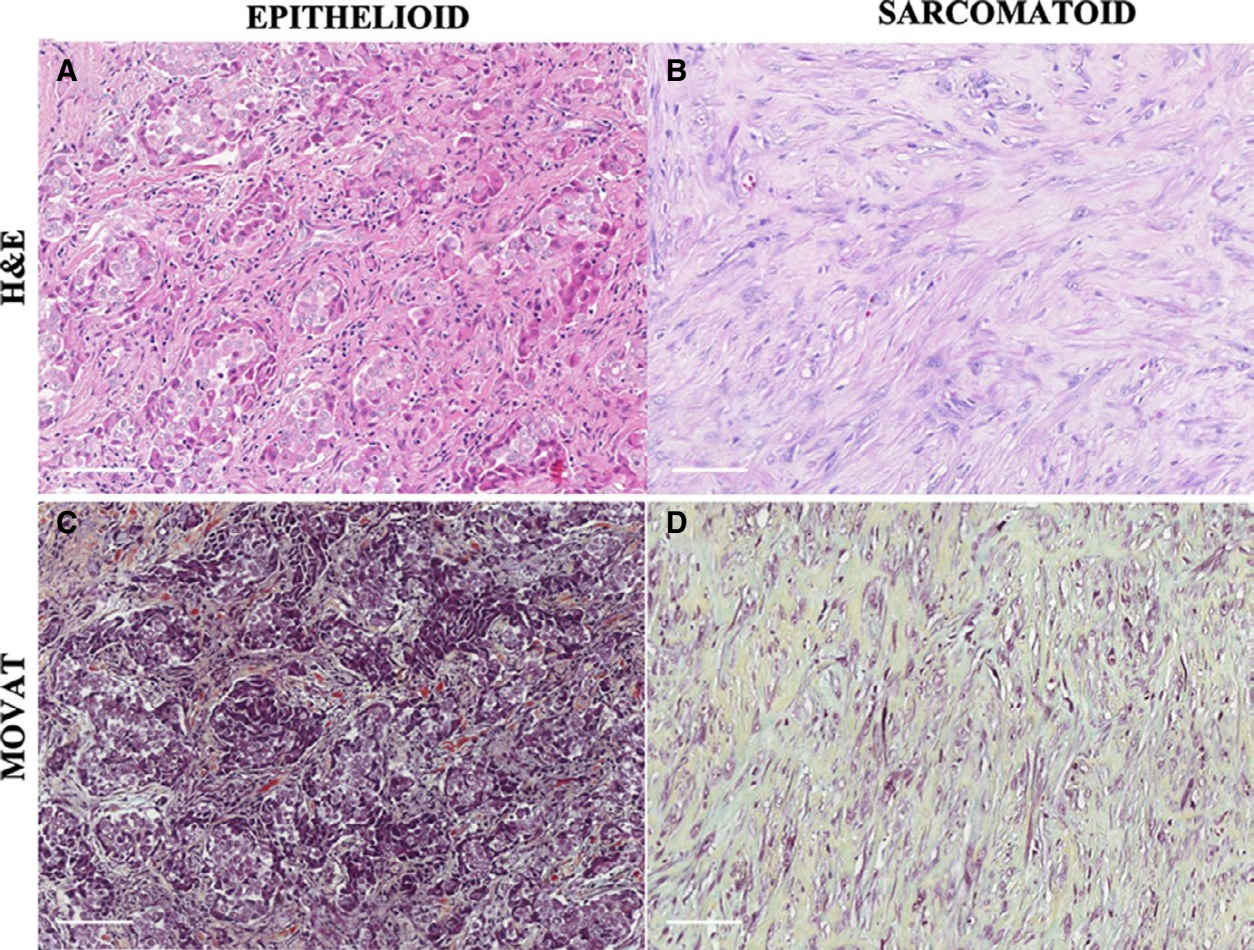
A representative epithelioid MM section showing blocks of epithelioid cells infiltrating a stromal element (A), correlated with Movat histochemical staining highlighting the matrix constituents surrounding neoplastic cells (C). A representative sarcomatoid MM section composed of malignant spindle cells contrasted by cellular densities and matrix‐forming elements, as explored with Movat staining (D)

**Figure 2:**
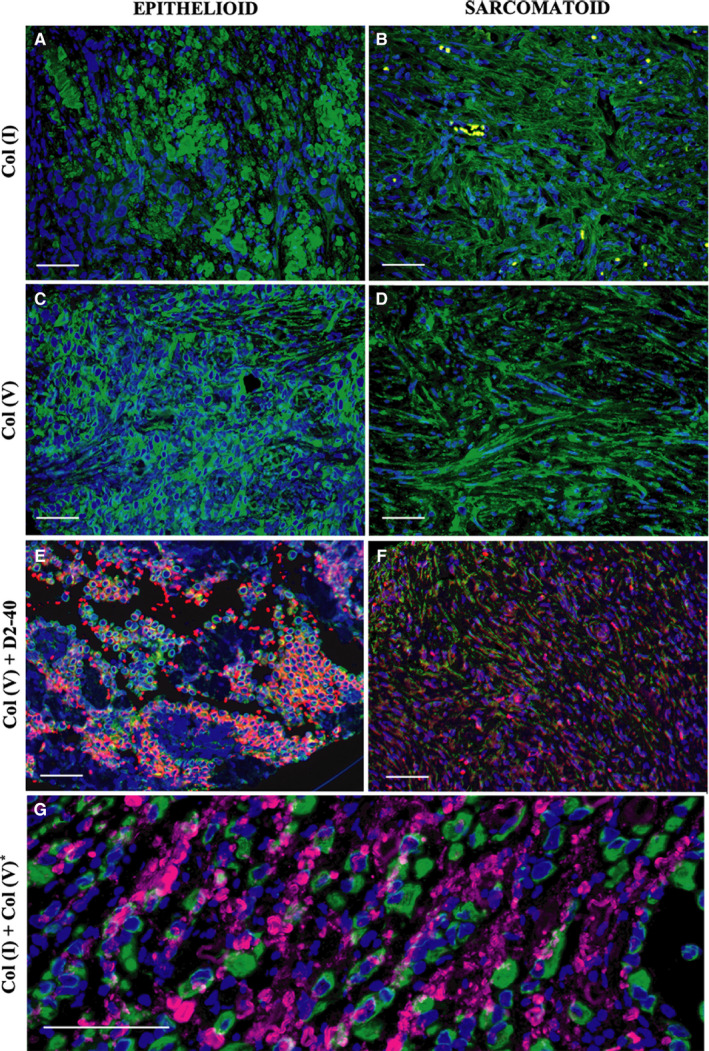
Immunofluorescence for collagen type I and type V in epithelioid and sarcomatoid subtypes of MM. Type I collagen shows a fibrillar pattern for both MM subtypes (A, B), while type V collagen surrounds the malignant cellular component (C, D). Double D2‐40 (red) and type V collagen (green) staining (E, F) show overlap of both stainings highly suggestive of deposition of collagen V in the membranes of tumor cells. A computer‐assisted collagen I and V image reconstruction is shown in G, highlighting the fibrillar (collagen I) vs the surrounding pattern of collagen V

**Figure 3:**
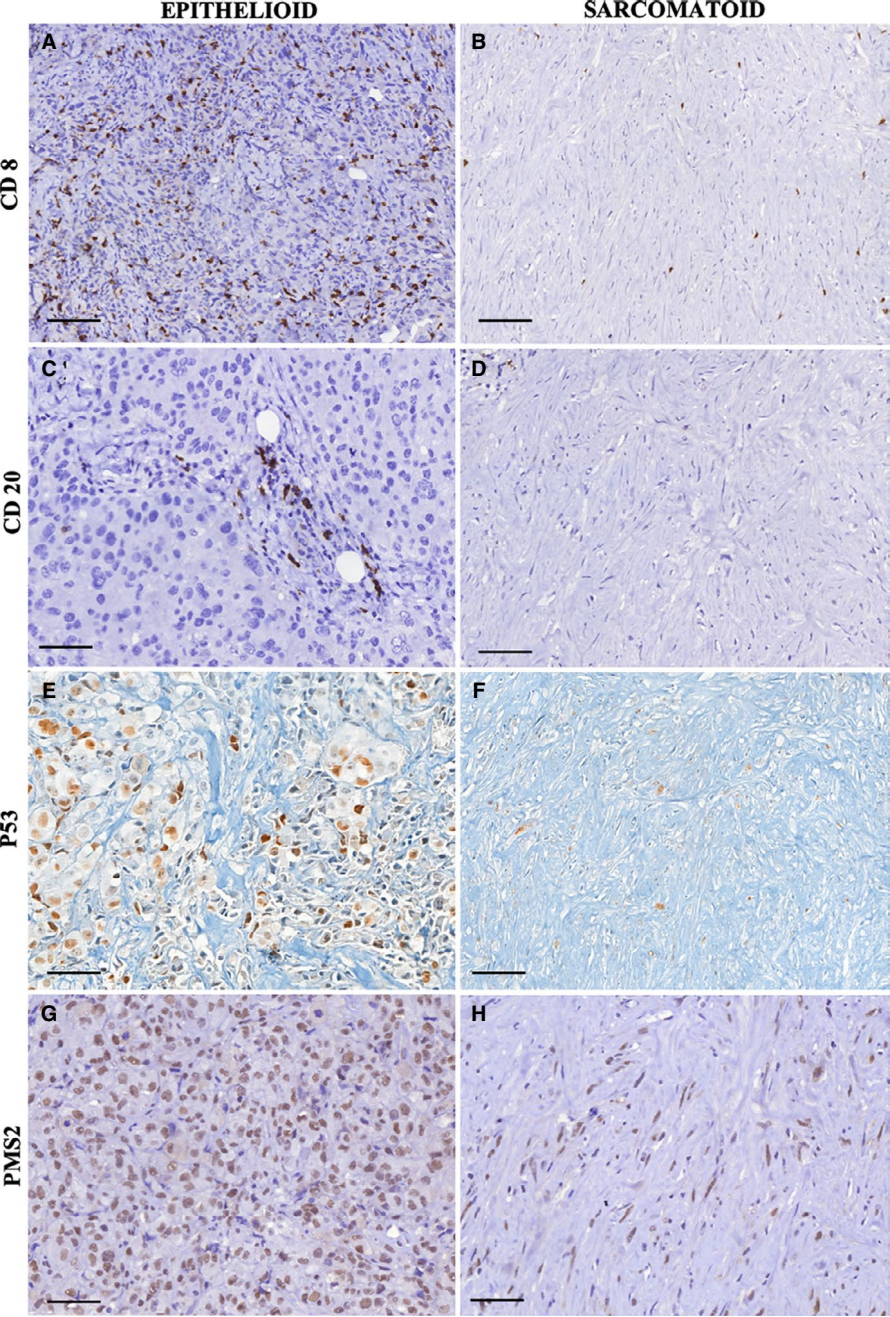
CD8^+^ T lymphocytes (A, B), CD2^+^ B lymphocytes (C, D), P53 immunoexpression (E, F), and PMS2 positivity (G, H) are compared between the epithelioid and sarcomatoid subtypes

**Figure 4:**
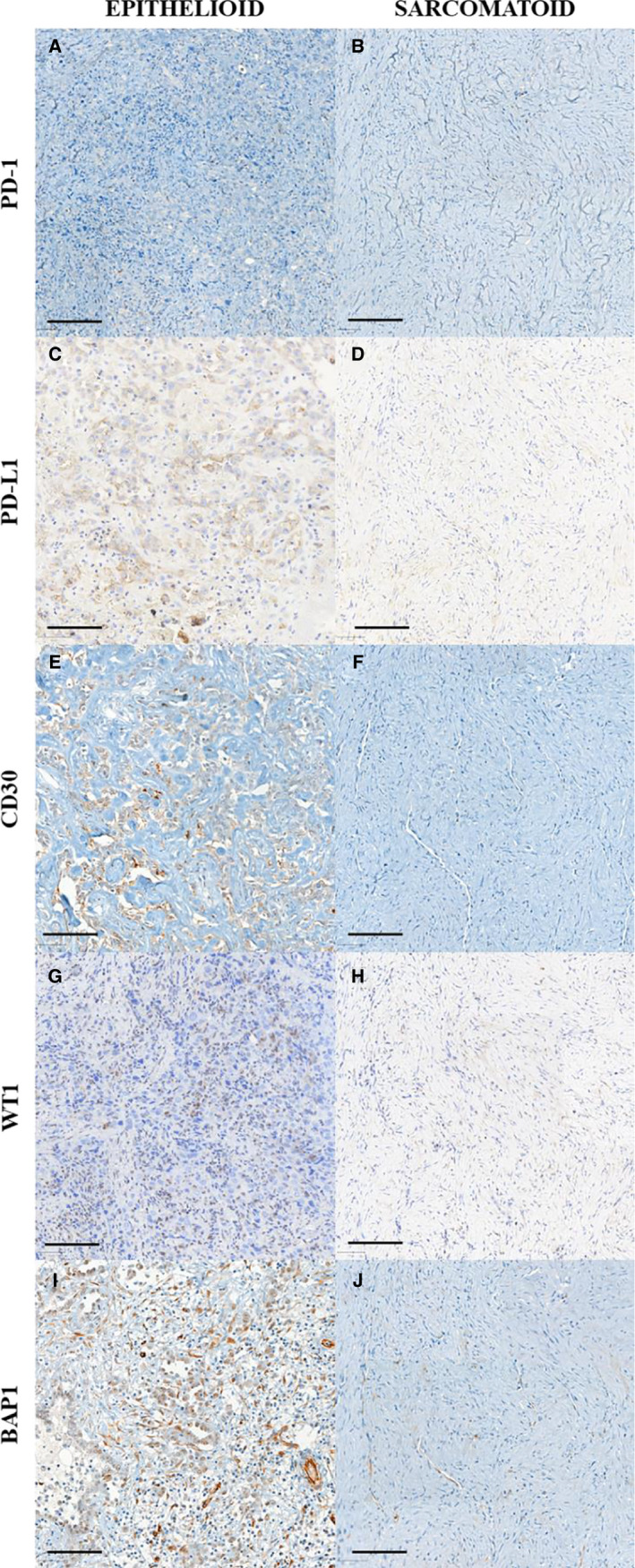
PD‐1 (A, B), PD‐L1 (C, D), CD30 (E, F), WT1 (G, H), and BAP1 (I, J) immunoexpression in epithelioid and sarcomatoid types

Epithelioid MM and sarcomatoid MM are represented in Figures 1, 2, 3, 4. Epithelioid MM shows high tumor cellularity (Figure 1A) with dense hyaluronic acid deposition in tumor stroma (Figure 1C). Epithelioid MM also exhibit a strong green birefringence of Col I (Figure 2A) and V (Figure 2C) fibers under fluorescence microscopy. In epithelioid MM, both collagen fibers are organized in a dense and irregular fibrillary pattern; however, Col I fibers surround groups of malignant cells, while Col V fibers involve individual malignant cells (Figure 2G). In epithelioid MM, CD8^+^ T lymphocytes (Figure 3A) are diffusely distributed and CD20^+^ B lymphocytes (Figure 3C) are patchy distributed. Malignant cells of epithelioid MM show strong expression of P53 (Figure 3E) and PMS2 (Figure 3G). Note sparse distribution of malignant cells from epithelioid MM positive for PD‐1 (Figure 4A), PD‐L1 (Figure 4C), CD30 (Figure 4E), WT1 (Figure 4G), and loss expression of BAP1 (Figures 4I). In contrast, sarcomatoid MMs have modest tumor cellularity (Figure 1B) and hyaluronic acid deposition (Figure 1D) as well as a distortion of the tumoral environment and a diffuse green birefringence of Col I (Figure 2B) and Col V (Figure 2D) fibers. In sarcomatoid MM, Col I fibers are organized predominantly in elongated thick fibers in individual large groups of sarcomatoid cells. Col V forms a more regular texture with thin fibers involving individual sarcomatoid cells coinciding with the fusiform pattern visualized under H&E staining (Figure 1B). Double staining for Col V and D2‐40 confirms the specificity of Col V expression surrounding MM cells (Figure 2‐E, F). Sparse CD8^+^ T lymphocytes (Figure 3B) and CD20^+^ B lymphocytes (Figure 3D) are found in the environment of sarcomatoid MM. The malignant cells in these tumors retain similar P53 expression (Figure 3F) and PMS2 (Figure 3H). Note the low expression of PD‐1 (Figure 4B), MC‐PD‐L1 (Figure 4D), CD30 (Figure 4F), WT1 (Figure 4H), and BAP1 (Figure 4J).

**Figure 5:**
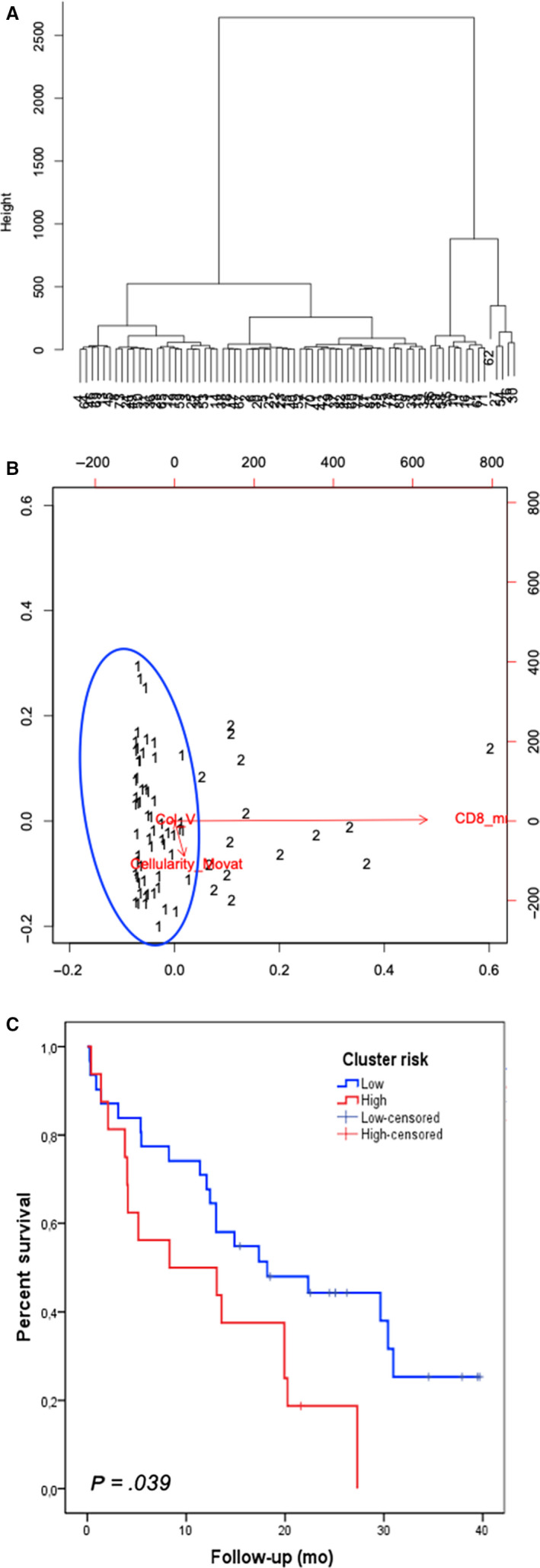
A, Dendrogram illustrating the arrangement of the clusters produced by the corresponding analyses of hierarchical clustering analysis of the studied 82 MM patients. Patients split into two clusters, which are separated by vertical lines. For markers, vertical lines on the left indicate low (below cutoff) expression or cellularity < 80.6 cells/mm^2^ CD4/CD8, collagen V < 7.75 fibers/mm^3^, and CD8 > 99.77 cells/mm^2^, whereas vertical lines on the right indicate high (above cutoff) expression or cellularity > 80.6 cells/mm^2^ CD4/CD8, collagen V > 7.75 fibers/mm^3^, and CD8 < 99.77 cells/mm^2^. Only significant (*P* < .05) discriminating markers or marker groups are annotated. B, Dispersion graph illustrates the grouping distribution of cases by the clustering model showing two risk groups: a distinct low‐risk cluster (grouping 1, blue circle) and a high‐risk cluster (grouping 2). C, Survival analysis of MM clusters. Note the poorer outcome of MM cluster 2 (linked to high expression of cellularity > 80.6 cells/mm^2^ CD4/CD8, higher expression of collagen V > 7.75 fibers/mm^3^, and lower expression of CD8 < 99.77 cells/mm^2^ compared with cluster 1; *P* = .039 (for comparison between all two clusters). MM indicates malignant mesothelioma; CD8 indicates cytotoxic lymphocytes

Table 2 summarizes the morphometric results of the tumors and surrounding microenvironment. Univariate analysis showed that epithelioid MM exhibit higher tumor cellularity, and more tumor cells expressing p53 protein and preserved PMS2 expression than sarcomatoid MM. Furthermore, epithelioid MM harbored significant more total collagen, Col I, and elastic fibers as well as CD8 T lymphocytes and CD20 B lymphocytes than sarcomatoid MM. No statistical differences were found for Ki‐67, TUNEL, MC‐PD‐L1, PD‐1, and repair proteins (MLH1, MSH2, and MSH6) between the two histotypes.

**Table 2:** Morphologic features and expression of various proteins as evaluated by immunohistochemistry according to histological subtype of malignant mesothelioma (n = 82)

	Epithelioid (n = 73)	Sarcomatoid (n = 9)	*P* value*
Tumor factors			
*Nuclear grade,* n* (%)*			
1	4 (4.9%)	2 (2.4%)	
2	45 (54.9%)	7 (8.5%)	.01
3	24 (29.3%)	0	
*Cellularity as mean # of cells/mm^2^*	71.14	53.22	<.005
*BAP‐1,* n* (%)*			
Loss of expression	56 (68.3%)	6 (7.3%)	.38
*P53* mean # of positive cells/mm^2^	2.74	0.66	<.005
*Ki‐67*	1.86	1.98	.89
*TUNEL*	20.20	14.48	.35
*MCs PD‐L1*	0.76	4.43	.32
*PD‐1*	0.45	0.35	.43
*MLH1*	48.87	35.22	.13
*MSH2*	47.92	41.28	.51
*MSH6*	55.85	52.80	.76
*PMS2*	41.99	23.35	.01
Microenvironment factors			
Hyaluronic acid mean density/mm^3^	6.57	10.73	.05
Elastin mean density/mm^3^	6.08	2.30	<.005
Total collagen fibers mean density/mm^3^	12.05	32.71	<.005
Collagen I mean density/mm^3^	1.10	0.16	<.005
Collagen V mean density/mm^3^	4.41	4.45	.97
CD4 mean # of cells/mm^2^	135.65	161.45	.83
CD8 mean # of cells/mm^2^	62.40	6.54	<.005
CD20 mean # of cells/mm^2^	110.02	5.79	<.005
CD68 mean # of cells/mm^2^	21.70	16.14	.26

Abbreviation: MCs, malignant cells.

* The Chi‐square test and *t* test were used to detect differences in categorical and continuous variables, respectively, between groups of patients. A *P* value ≤ .05 was considered statistically significant.

The morphometric variables that differed significantly between epithelioid and sarcomatoid MM by univariate analysis (nuclear grade, tumor cellularity, p53 protein, PMS2 mismatch repair protein, hyaluronic acid, elastic fibers, total collagen, Col I, Col V, and CD8^+^ T lymphocytes) were grouped by hierarchical cluster analyses independent of clinicopathological variables; two clusters of patients with 67 subjects in cluster 1 (CL I) and 15 in cluster 2 (CL II) were identified (Figure 5A,B; Table 3). Figure 5A shows the cluster dendrogram, separating two groups by dispersion similarities, as plotted in the scatterplot (Figure 5B). CL I, categorized as low risk for death of disease, includes tumors with a cellularity of less than 80.6 cells/mm^2^, Col V fiber density of less than 7.75 fibers/mm^3^, and CD8 T lymphocyte mean cellularity of more than 99.77 cells/mm^2^. CL II, categorized as high risk, is comprised of MM with a cellularity of more than 80.6 cells/mm^2^, Col V fiber density of more than 7.75 fibers/mm^3^, and CD8 T lymphocyte cellularity of less than 99.77 cells/mm^2^.

**Table 3:** Hierarchical cluster analysis identified two clusters of patients with malignant mesothelioma

Cluster I Low risk	Cluster II High risk
Cellularity < 80.6 cells/mm^2^	Cellularity > 80.6 cells/mm^2^
Collagen V < 7.75 fibers/mm^3^	Collagen V > 7.75 fibers/mm^3^
CD8 > 99.77 cells/mm^2^	CD8 < 99.77 cells/mm^2^

The results of the multivariable analysis are summarized in Table 4. Multivariate analysis shows that tumor cellularity > 80.6 cells/mm^2^, Col V > 7.75 fibers/mm^3^, and CD8 T lymphocytes < 99.77 cells/mm^2^ are associated with an increased risk of death. Patients with MM in CL II had twice the risk of death than patients in CL I.

**Table 4:** Multivariate analysis of studied variables in malignant mesothelioma

	Risk of death	*P* value
Variable	OR (95% CI)	
Cellularity >80.6 cells/mm^2^	1.63 (0.45‐2.89)	.02
Col V >7.75 fibers/mm^3^	2.60 (0.98‐3.33)	.04
CD8 <99.77 cells/mm^2^	1.001 (0.995‐1.007)	<.005
Cluster analysis		
Low risk	0.48 (0.12‐1.84)	<.005
High risk	2.19 (0.54‐3.03)	.006

Results of univariate and multivariate analyses of clinicopathologic variables in relationship to risk of death at 40 months are summarized in Table 5 **.** Asbestos exposure, biopsy (vs surgical resection), and high cluster group are associated with increased risk of death at 40 months in univariate analysis. Biopsy (vs surgical resection) and high cluster group continued to be significant for risk of death at 40 months in the multivariate analysis; no chemotherapy became significant while asbestos exposure was not associated with outcome anymore. Whereas the overall likelihood ratio of the Cox model using asbestos exposure, histotype, and treatment alone was only 12.42, the likelihood ratio with treatment and cluster groups was 15.05. Figure 5C shows the Kaplan‐Meier curves of the high‐risk and low‐risk clusters for all MM. The patients in the low‐risk cluster CL I had a better overall survival with a median survival time that was not reached during follow‐up and a mean survival time of 21 months. In contrast, patients in the high‐risk cluster CL II had a shorter median survival time of only 12 months (*P* = .03).

**Table 5:** Hazard ratios (HRs) of clinicopathological characteristics for risk of death at 40 mo for patients with malignant mesothelioma

Variable	Univariate analysis HR (95% CI)	*P* value	Multivariate analysis HR (95% CI)	*P* value
*Cluster*				
High risk vs Low risk	1.32 (0.68‐2.58)	.01	1.28 (0.60‐2.75)	.01
*Asbestos exposure*				
Yes vs No	1.88 (1.004‐3.52)	.04	1.02 (0.51‐2.05)	.95
*Histologic type*				
Sarcomatoid vs Epithelioid	1.12 (0.46‐2.72)	.05	1.86 (0.69‐5.007)	.21
*Treatment*				
Surgical resection				
No vs Yes	3.04 (1.37‐6.74)	.006	2.83 (1.14‐7.009)	.02
Chemotherapy				
No vs Yes	2.41 (0.85‐6.84)	.05	3.47 (1.01‐11.83)	.04

## 4 DISCUSSION

In this study, we found a variety of associated effects on the behavior of MM, including the following: (a) the clinicopathological characteristics of our cohort were in agreement with other studies; (b) an objective quantitative approach to evaluate tumor and microenvironment biomarkers was established; (c) validation of established clustering as the grouping of objects by calculating similarities in high‐throughput protein analyses was performed to characterize two subgroups of MM risk; (d) Col V, CD8^+^ T lymphocytes, and tumor cellularity densities characterized high‐ and low‐risk subgroups with impact on outcome, independent of the histologic grade, classification, and other tumor factors; and (e) a low mutational burden and the tumor microenvironment rather than genetic abnormalities in MM cells contributed to MM asbestos‐related progression. To the best of our knowledge, this was the first study to analyze the associations between the potential effect of a quantitative approach for immune profiling and the matricial modulatory control of MM behavior.

Our cohort included 82 MM patients. The clinicopathological characteristics of the patients concur with previous studies concerning clinicopathological characteristics, including age, sex, asbestos exposure, histologic subtypes,^3^ site of MM,^1^ stage,^5^ and survival.^4^, ^6^ Although surgical resection was performed in the majority of our patients followed by adjuvant chemotherapy, 49 (56%) patients died of disease. Therefore, risk stratification of patients with MM using prognostic and predictive biomarkers is important.^32^ Despite advances in our understanding of the pathogenesis of MM, there are complex challenges in identifying such biomarkers. One of the major problems is the lack of adequate adjustment for potential confounders. For instance, the reporting of biomarkers differs greatly between studies with some using binary cut‐points to define a positive tumor or reporting results in a semiquantitative fashion and others expressing staining in relation to stromal staining or expression of other proteins.

From this study, using an artificial intelligence quantitative approach, we evaluated the association between tumor and tumor microenvironment and the behavior of MM in these patients. Univariate analysis showed that epithelioid MM presented with high cellularity and expression of p53 protein and retained expression of PMS2 protein, while the environment showed high amounts of total collagen, Col I, elastic fibers, and high numbers of CD8^+^ T lymphocytes. In contrast, sarcomatoid MM tumors showed lower cellularity, no p53 expression, and lack of expression of PMS2 protein, together with increased total collagen fibers. Col V was similarly present around mesothelial tumor cells coinciding with hyaluronic acid expression in both histologic types. The presence of p53 expression and preserved expression of PMS2 protein indicates a low mutational burden, while collagen fibers and immune cells compose a rich microenvironment rather than genetic abnormalities in MM cells, which may contribute to MM progression.

Subsequently, these variables that were significantly different between epithelioid and sarcomatoid MM and independent of clinicopathological variables were grouped by calculating similarities in high‐throughput protein analyses for characterizing different subgroups of MM. This analysis identified two subgroups of patients composing CL I (low risk of death) and CL II (high risk of death). Overall, our results showed that the risk of death was increased for patients in cluster II that was characterized by increased tumor cellularity, denser Col V fibers, and less CD8 T lymphocytes. In fact, these patients had two times the risk of death than patients in CL I.

Our CL II indicates that Col V is present in increased amounts in the ECM microenvironment of MM. We have shown that in epithelioid MM, Col I fibers surround groups of malignant cells, while Col V fibers surround individual malignant cells. In contrast, in sarcomatoid MM, Col I fibers show a different pattern and are organized as elongated thick fibers involving large groups of tumor cells in a radial alignment, while Col V fibers form a more regular texture with thin fibers involving individual malignant fusiform cells. In particular, local cell invasion has been found to be predominantly oriented along certain aligned collagen fibers, suggesting that the radial alignment of Col fibers relative to tumor cells facilitates invasion.^33^ The reappearance of the “embryonic” Col I‐trimer and an increase in Col V content in the ECM of breast carcinoma have been postulated.^34^ Luparello et al^34^ compared the effects of these two collagen types with Col I as culture substrata on the spreading pattern, cytoskeletal organization, and motile behavior of cultured breast carcinoma cells. They found that cells grown on Col I were stationary, with a well‐spread morphology and an extensive stress fiber pattern, while cells grown on Col V were also stationary but exhibited a poorly spread and elongated morphology. In contrast, cells grown on Col I‐trimer were motile and displayed a compact arrangement and a reduced content of stress fibers. Both single‐cell and group motility were detectable on trimer Col substratum. These data are consistent with the existence of two opposite local signals, Col I‐trimer and Col V, which may confer different metastatic phenotypes in breast carcinoma cells. In other words, the re‐deposition of “embryonic” Col in ECM, produced by the tumor cells themselves, may be involved in stromal pathways that allow the neoplastic cells to infiltrate the surrounding host tissue, by analogy with the “contact guidance” role of the ECM substrata during development.

Our present results raise questions concerning the origin of increased Col V. We presented evidence suggesting that the source of Col V in the environment is related to invasive tumor cells. Immunofluorescence data consistently revealed the presence of Col V involving the membrane of invasive tumor cells. The pattern of Col V in the stroma as fibrillar and linear deposits, in contrast to the more diffuse homogeneous distribution of Col I, emphasizes the unique distribution properties of Col V as a pericellular interstitial collagen in the ECM environment. Our observations of the presence of Col V involving the tumor cells are different from the observations of other investigators in other carcinomas, including breast carcinoma. These carcinomas consistently synthesized Col V, which occurred as linear deposits in the interstitium (desmoplastic stroma surrounding the tumor).^33^, ^35^ We were able to demonstrate pericellular membranous staining of Col V with our immunofluorescence technique. We therefore hypothesized that the increased Col V observed in the ECM microenvironment represented a host response to invasive MM and that this collagen was synthesized by MM cells. While the precise origin of Col V is, in general, still controversial, Col V is thought to be present adjacent to the basement membranes of mesothelial cells (MCs) and occur as a pericellular collagen. In fact, MCs are epithelial‐like cells that are embryologically derived from the mesoderm and express mesenchymal features, including vimentin and desmin.^36^ MCs are pluripotent cells capable of synthesizing cytokines/chemokines, growth factors, ECM molecules (including collagen types I, III, and IV), elastin, fibronectin, laminin, and proteoglycans.^19^, ^37^, ^38^, ^39^, ^40^, ^41^ Therefore, we hypothesized that MCs have the ability to synthesize Col V. Our present observation of increased Col V in the ECM microenvironment raises the possibility that infiltrating MM stimulates malignant cells to secrete Col V. Our observations of increased Col V in MM are similar to previous findings in breast cancer,^35^ colorectal cancer,^42^ lung cancer,^43^ and primary pancreatic ductal carcinoma.^44^

Col V has also been reported as an autoantigen in chronic inflammatory diseases,^45^ acute and chronic lung transplants,^46^ and lung cancer.^25^ As a minor collagen in the ECM, Col V, is normally sequestered within the ECM and is therefore hidden from the immune system. ECM remodeling induced by malignant cells may cause Col V exposure and overexpression, which may in turn lead to the development of anti‐Col V immunity facilitating tumor progression. Additionally, in antitumor immunity, CD8^+^ cytotoxic T‐lymphocytes and natural killer cells play a role as effectors, inducing tumor cell apoptosis^47^ and thus inhibiting tumor growth.^12^ Furthermore, previous reports suggest that asbestos might gradually impair antitumor immunity.^48^ Kumagai‐Takei and colleagues^49^ demonstrated that MM patients present features of impairment in the stimulation‐induced cytotoxicity of peripheral blood CD8^+^ T lymphocytes, possibly related to asbestos exposure. Moreover, asbestos exposure is associated with the production of MC autoantibodies and induces collagen deposition by cultured MCs.^11^ In our cohort of patients exposed to asbestos, we also showed that low CD8^+^ T lymphocyte infiltration in the MM environment was associated with aggressive behavior, in agreement with previous reports.^50^, ^51^

Thus, for all the above reasons, we should not be surprised to learn that combining immune matricial factors may provide important prognostic information about MM. Our results have confirmed the prognostic importance of Col V and CD8^+^ T lymphocytes in this malignancy. In the Cox regression analysis, we found that asbestos exposure, sarcomatoid subtype, biopsy (vs surgical resection), and adjuvant chemotherapy were significantly related with increased risk of death in the absence of cluster groups; moreover, when cluster groups were present as covariates, their relationship to increased risk of death was much stronger.

Our analysis has a number of limitations that could not be addressed in this first quantitative study. A major limitation was the relatively small cohort of sarcomatoid MM (N = 9; 11%) retrospectively collected cases with a maximum follow‐up of 40 months. Another limitation was the TMA format, which could have induced under‐ or overrepresentation of variables due to intratumoral heterogeneity. Furthermore, we lacked data regarding responses to immune checkpoint inhibitor therapies in our patient population. In the future, we hope to be able to perform similar studies on samples from patients treated with such therapy.

We conclude that in our cohort, asbestos‐related MMs were characterized by a low mutational burden and a distinct tumor microenvironment rather than genetic abnormalities. Col V fiber density and CD8^+^ T lymphocytes in tumors with high cellularity might be potential targets for therapies that control tumor growth rate, motility, and invasion in MM.

## CONFLICT OF INTEREST

The authors have disclosed that they have no significant relationships with, or financial interest in, any commercial companies pertaining to this article.

## AUTHORS CONTRIBUTION

Marcelo Balancin: manuscript draft, data collection, computational analysis curation, case review and statistical analysis. Walcy Rosolia Teodoro: immunofluorescence validation and performance, study design input, and manuscript draft. Cecilia Farhat: statistical analysis. Tomas Jurandir de Miranda: immunofluorescence quantification. Aline Kawassaki Assato: tissue microarray construction. Neila Aparecida de Souza Silva: immunohistochemistry validation and performance. Ana Paula Velosa: immunofluorescence validation and performance. Roberto Falzoni: immunohistochemistry validation and performance and study design input. Alexandre Muxfeldt Ab’Saber: material review. Anja C. Roden: immunohistochemistry validation and performance, study design input, and critical review of the manuscript. Vera Luiza Capelozzi: manuscript draft, study design input, statistical analysis, and case review.

## Supporting information

Table S1

## Data Availability

The data that support the findings of this study are available from the corresponding author upon reasonable request due to ethical restrictions from local legislation.
