# Supplementary material for: An integrative histopathologic clustering model based on immuno‐matrix elements to predict the risk of death in malignant mesothelioma
Source: Cancer Med. 2020 May 11;9(13):4836–49. doi: 10.1002/cam4.3111 (PMC7333849; doi:10.1002/cam4.3111)
Supplement: Supplementary file 1 — Table S1 [file CAM4-9-4836-s001.docx]

| **Antigen** | **Clone** | **Source** | **Working Dilution** | **Citrate Buffer (pH)** | **Antigen retrieval** | **Detection System** | **Method** |
| --- | --- | --- | --- | --- | --- | --- | --- |
| CD4 | 368 | Novocastra | 1:50 | 9,0 | Steamer | Reveal | Manual |
| CD8 | C8/144B | Dako | 1:400 | 6,0 | Steamer | Reveal | Manual |
| CD20 | L26 | Dako | 1:2000 | 6,0 | Steamer | Reveal | Manual |
| CD68 | KP1 | Dako | 1:5000 | 6,0 | Steamer | Reveal | Manual |
| D2-40 | D2-40 | Dako | 1:1000 | 6,0 | Steamer | Reveal | Aut.^*^ |
| WT-1 | 6F-H2 | Cellmarque | Prediluted | 8,4 | Ultra CC1 | Ultraview | Aut. |
| MOC31 | MOC31 | Roche | Prediluted | 8,4 | Ultra CC1 | Ultraview | Aut. |
| Ber-EP4 | Ber-EP4 | Cellmarque | Prediluted | 8,4 | Ultra CC1 | Ultraview | Aut. |
| BAP1 | C-4 | Santa Cruz | Prediluted | 8,4 | Ultra CC1 | Optiview | Aut. |
| P53 | DO-7 | Roche | Prediluted | 8,4 | Ultra CC1 | Ultraview | Aut. |
| Ki-67 | MIB-1 | Dako | 1:400 | 6,0 | Steamer | Novolink | Aut. |
| PD-1 | Nat 105 | Cellmarque | Prediluted | 8,4 | Ultra CC1 | Ultraview | Aut. |
| PD-L1 | SP263 | Roche | Prediluted | 8,4 | Ultra CC1 | Optiview | Aut. |
| CD30 | BER-H2 | Roche | Prediluted | 8,4 | Ultra CC1 | Optiview | Aut. |
| MLH1 | M1 | Roche | Prediluted | 8,4 | Ultra CC1 | Optiview | Aut. |
| MSH2 | G219-1129 | Roche | Prediluted | 8,4 | Ultra CC1 | Optiview | Aut. |
| MSH6 | SP93 | Roche | Prediluted | 8,4 | Ultra CC1 | Optiview | Aut. |
| PMS2 | A16-4 | Roche | Prediluted | 8,4 | Ultra CC1 | Optiview | Aut. |

**Supplementary Table 1**. Primary antibodies, clones, dilution and source used.

*Automatized technique, Ventana Benchamark Ultra
